# Ekbom’s syndrome in an HIV man: a case report

**DOI:** 10.1192/j.eurpsy.2022.1510

**Published:** 2022-09-01

**Authors:** D. Paiva Pajares, C. Hernández Peláez, A. Martínez Muelas, M. López Isern, T. Castelló Pons

**Affiliations:** 1 Hospital Sagrat Cor, Hermanas Hospitalarias., Psychiatry, Martorell (Barcelona), Spain; 2 Hospital Clínico San Carlos, Intensive Care Unit, Madrid, Spain

**Keywords:** psychiatry, Ekbom, HIV, emergency

## Abstract

**Introduction:**

Ekbom’s syndrome, also known as delusional parasitosis, is a neuropsychiatric disorder characterized by the delusional belief that the body is infested by parasites, small organisms or materials. Multiple etiologies have been described such as psychiatric and neurological disorders, substance intoxication or other medical conditions. We present a case of Ekbom’s syndrome in an individual infected with the human immunodeficiency virus (HIV).

**Objectives:**

To report a case of a patient with Ekbom’s syndrome and HIV.

**Methods:**

A 33-years-old man assists to the emergency unit in order to excessive drowsiness. During the evaluation an antihistamin overdose is confirmed. The patient justifies taking it by claiming to have parasites all over the skin, a fact that is ruled out. Medical history is reviewed presenting multiple visits to GP for thinking that he has parasites, performing medical examinations without alterations. Toxicological, hemogram, biochemistry, hormonal and vitamin study did not show alterations.

**Results:**

Due to the symptoms presented, it was decided to start antipsychotic therapy. At the beginning, the patient is not aware of needing treatment other than antiparasitic. After optimizing the olanzapine dose to reach 20 mg / day, the patient denied experiencing tactile and visual hallucinations.

**Conclusions:**

Ekbom’s syndrome is a multifactorial neuropsychiatric disorder, individuals infected with HIV are at increased risk of psychotic disorders. The patient was diagnosed of psychotic disorder due to another medical condition because the history of HIV preceded the history of delusional content.

**Disclosure:**

No significant relationships.

